# Supplementary figures and images for: Gamma-Tocotrienol Protects the Intestine from Radiation Potentially by Accelerating Mesenchymal Immune Cell Recovery
Source: Antioxidants (Basel). 2019 Mar 6;8(3):57. doi: 10.3390/antiox8030057 (PMC6466604; doi:10.3390/antiox8030057)

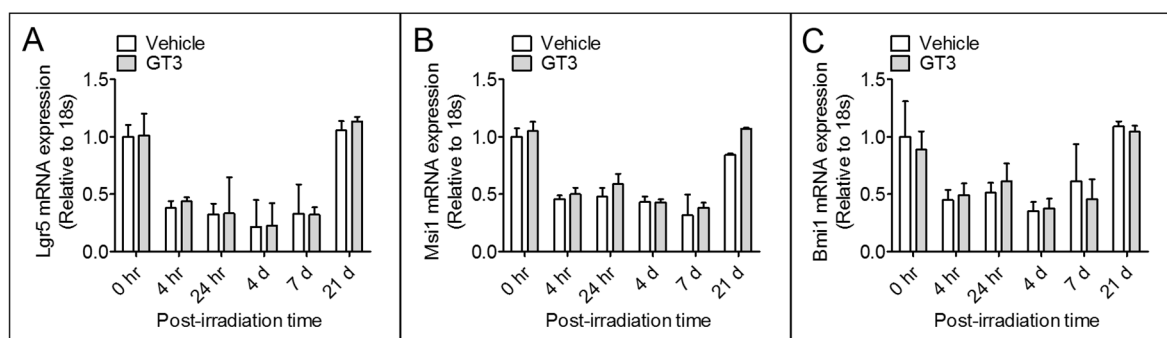

**Figure S1.** Effects of GT3 pretreatment on TBI-induced intestinal stem cell markers.

Supplement: Supplementary file 1 [file antioxidants-08-00057-s001.pdf]
